# Gestational age-specific serum creatinine can predict adverse pregnancy outcomes

**DOI:** 10.1038/s41598-022-15450-w

**Published:** 2022-07-02

**Authors:** Jieun Kang, Sangwon Hwang, Tae Sic Lee, Jooyoung Cho, Dong Min Seo, Seong Jin Choi, Young Uh

**Affiliations:** 1 Department of Obstetrics and Gynecology, Wonju Severance Christian Hospital, Yonsei University Wonju College of Medicine, 20 Ilsan-ro, Wonju, 26426 Republic of Korea; 2 Artificial Intelligence Bigdata Medical Center, Yonsei University Wonju College of Medicine, Wonju, Republic of Korea; 3 Department of Family Medicine, Yonsei University Wonju College of Medicine, Wonju, Republic of Korea; 4 Department of Laboratory Medicine, Wonju Severance Christian Hospital, Yonsei University Wonju College of Medicine, 20 Ilsan-ro, Wonju, 26426 Republic of Korea; 5 Department of Medical Information, Yonsei University Wonju College of Medicine, Wonju, Republic of Korea

**Keywords:** Medical research, Nephrology, Risk factors

## Abstract

Serum creatinine level (SCr) typically decreases during pregnancy due to physiologic glomerular hyperfiltration. Therefore, the clinical practice of estimated glomerular filtration rate (eGFR) based on SCr concentrations might be inapplicable to pregnant women with kidney disease since it does not take into account of the pregnancy-related biological changes. We integrated the Wonju Severance Christian Hospital (WSCH)-based findings and prior knowledge from big data to reveal the relationship between the abnormal but hidden SCr level and adverse pregnancy outcomes. We analyzed 4004 pregnant women who visited in WSCH. Adverse pregnancy outcomes included preterm birth, preeclampsia, fetal growth retardation, and intrauterine fetal demise. We categorized the pregnant women into four groups based on the gestational age (GA)-unadjusted raw distribution (Q1–4_raw_), and then GA-specific (Q1–4_adj_) SCr distribution. Linear regression analysis revealed that Q1-4_adj_ groups had better predictive outcomes than the Q1–4_raw_ groups. In logistic regression model, the Q1–4_adj_ groups exhibited a robust non-linear U-shaped relationship with the risk of adverse pregnancy outcomes, compared to the Q1–4_raw_ groups. The integrative analysis on SCr with respect to GA-specific distribution could be used to screen out pregnant women with a normal SCr coupled with a decreased renal function.

## Introduction

Pregnancy is known to cause physiological alterations, including blood volume expansion via increased cardiac output and decreased systemic vascular resistance^1,2^. Specifically, systemic vasodilation and glomerular hyperfiltration are normal hemodynamic adaptations in pregnancy^1^. As gestational age (GA) increases, the glomerular filtration rate (GFR) during pregnancy increases physiologically, and the serum creatinine level decreases^3^. The currently used GFR estimation formula based on blood creatinine concentrations cannot be accurately applied in pregnant women^4,5^.

The Modification of Diet in Renal Disease (MDRD) formula^4^, which estimates GFR using a combination of serum markers and clinical parameters, has become a standard clinical method to estimate renal function in patients with chronic kidney disease (CKD). The feasibility of using this formula in the pregnant population has not been well studied, and guidelines on application of the MDRD formula specifically exclude interpretation in pregnant women^5,6^. Hence, it can be interpreted as a normal reference range for pregnant women with abnormal kidney function because it does not reflect normal physiological changes during pregnancy^5^. This makes it difficult to diagnose early and provide proper treatment for complications that can affect both fetuses and mothers^7^.

The incidence of CKD increases with pregnancy at advanced maternal ages^2^. Women with renal disorders face several problems in pregnancy due to increased physiologic changes accompanying renal dysfunction, including risk for disease progression, the potential teratogenicity of medications, and the increased risk of complications such as preeclampsia and preterm delivery^2,8–10^. Therefore, it is important to accurately evaluate renal function during pregnancy^11^.

Pregnancy status in CKD patients is known to be related with adverse pregnancy outcomes, such as pre-eclampsia^12^ and premature delivery^13,14^. Pregnant women diagnosed with CKD were typically categorized into three groups based on the severity of kidney dysfunction: mild (SCr < 1.5 mg/dL), moderate (1.5–2.5 mg/dL), and severe (> 2.5 mg/dL)^14^. However, Smith et al.^5^ suggested that the sole application of SCr concentration or SCr-based equations tend to substantially underestimate renal function during pregnancy. The Torino-Cagliari Observational Study^15^ found that the risk of adverse pregnancy outcomes increases stepwise as CKD stage 1 transitions to stages 4–5. However, the risk of adverse pregnancy outcomes was not significantly different between pregnant CKD stage 1 patients and normal pregnant women^5^. Multidisciplinary expert groups in Seoul National University focused on GFR estimated by midterm SCr to identify the association between kidney function and adverse pregnancy outcomes^9^.

Our study had two main tasks. First, we integrated an automatic platform and a manual method to establish a dataset on pregnant women. The automatic work was performed by a laboratorian, a computer scientist, and a database administrator, and the manual task was performed by domain experts, including an obstetrician and a family medicine doctor. Second, we implemented prior knowledge^16^ to improve the predictive power of adverse pregnancy outcomes. Recently, Harel, Ray and their colleagues^8,16^ collected approximately 362,000 measurements of SCr levels from 243,534 women, and established the GA-specific distribution. In this study, we applied the previously validated findings to identify the association between kidney function and adverse pregnancy outcomes.

## Results

### Determination of SCr group based on the GA-specific SCr distribution

**Figure 1:**
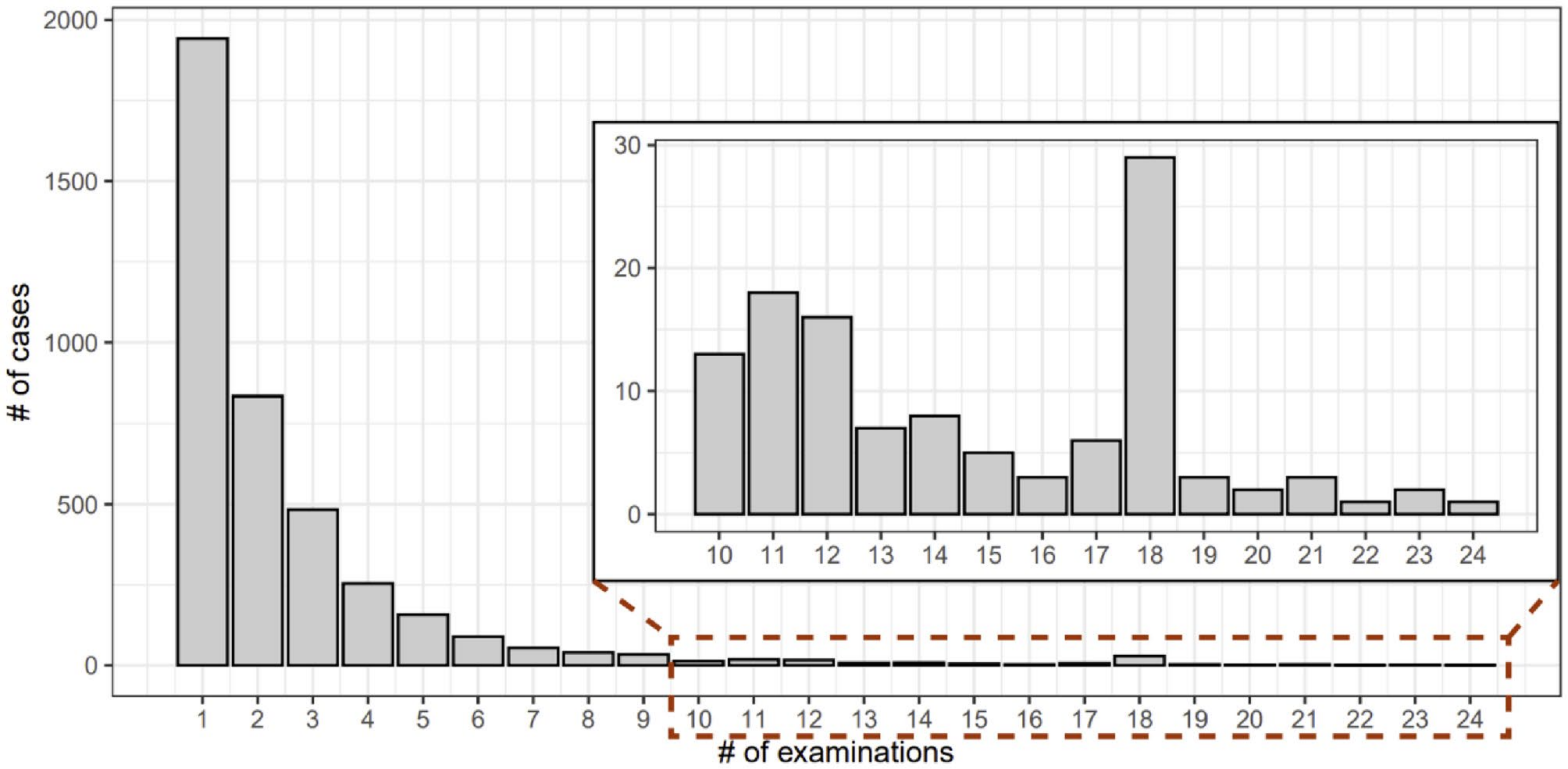
The number of cases according to the number of examinations performed to determine serum creatinine levels.

We analyzed 4004 pregnant women who visited Wonju Severance Christian Hospital (WSCH) during the study period. For the 4004 subjects, 10,126 measurements of SCr levels existed, indicating that a person had an average of 2.53 times of SCr evaluations. In most cases (n = 1942), the SCr was measured once; it was measured 23 times in two cases and 24 times in one case (Fig. 1). For patients with multiple SCr measurements, we used the maximum estimated value of SCr level.

**Figure 2:**
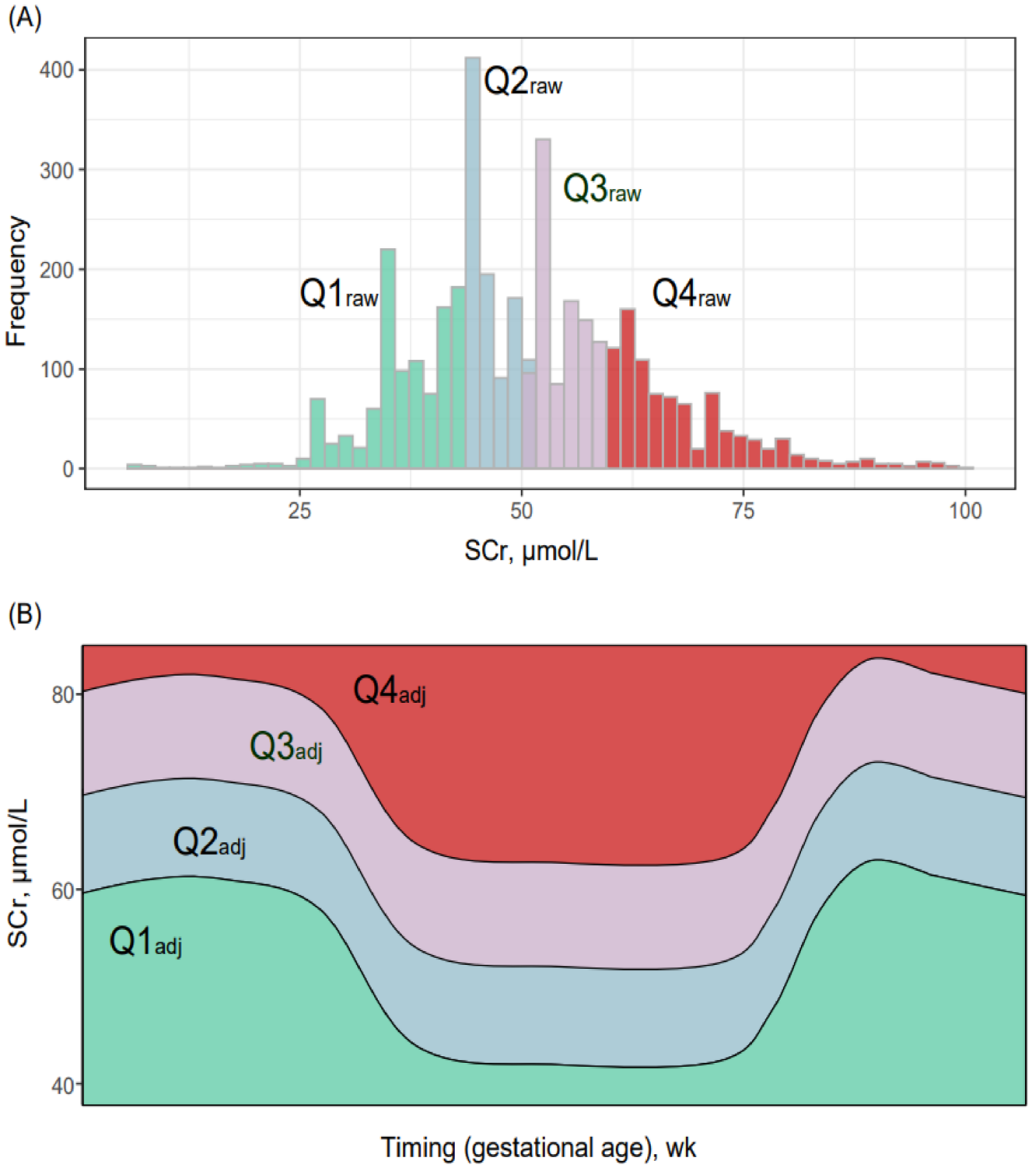
Figure shows the two methods used to categorize the subjects into four groups based on the levels of serum creatinine (SCr). (**A**) Figure shows the distribution of our original or raw data. (**B**) Figure shows the abstract version of the distribution of gestational age-specific serum creatinine made by Harel et al^16^.

We used two methods for grouping all pregnancies based on the level of SCr (Fig. 2). At first, we divided SCr into four groups based on the 25th, 50th, and 75th percentile of SCr levels (Fig. 2A), thereby, 1097, 978, 955, and 974 subjects were evenly grouped into four raw groups by SCr levels [quartile (Q)1_raw_ to Q4_raw,_ respectively]. For the other method, we grouped all SCr results according to the distribution of GA-specific SCr levels, as proposed by Harel and colleagues^16^. In detail, we mapped our SCr results onto this distribution that includes GA and SCr levels as X and Y axes, respectively (Fig. 2B). As a result, 1,888, 813, 835, and 468 subjects were arranged into four groups [Q1_adj_ (an adjusted group by GA-specific Scr distribution) to Q4_adj_, respectively].

### Medical and biochemical characteristics

General characteristics, including medical and biochemical data based on the four SCr groups determined by the GA-specific SCr distribution^16^ are summarized in Table 1. The average SCr levels showed a stepwise increase as it transitioned from the Q1_adj_ to Q4_adj_ group. The mean age was lowest in Q1_adj_ group, but did not significantly differ among Q2_adj_ to Q4_adj_ groups (Table 1). The subjects in the Q4_adj_ group had the following characteristics when compared to the Q1_adj_ group: high ratio of multi-para, diabetes, preterm birth (PTB), preeclampsia, and fetal growth retardation (FGR) (Table 1).

**Table 1:** Medical and biochemical characteristics based on SCr groups categorized by the gestational age-specific serum creatinine distribution made by Harel and colleagues^16^.

	Q1_adj_	Q2_adj_	Q3_adj_	Q4_adj_	*P* for trend
n	1,888	813	835	468	
SCr, μmol/L	41 ± 0.17	52.8 ± 0.17	60.1 ± 0.2	86.2 ± 3.1	< 0.001
**SCr raw groups, n**					
Q1_raw_	1097 (58.1)	0 (0)	0 (0)	0 (0)	
Q2_raw_	663 (35.1)	298 (36.7)	17 (2)	0 (0)	
Q3_raw_	121 (6.4)	423 (52)	402 (48.1)	9 (1.9)	
Q4_raw_	7 (0.4)	92 (11.3)	416 (49.8)	459 (98.1)	
Age, year	32.6 ± 0.12	33.2 ± 0.17	33.6 ± 0.18	33.6 ± 0.23	< 0.001
**Parity type**					
Nulliparity, n	974 (51.6)	361 (44.4)	324 (38.8)	165 (35.3)	< 0.001
Multiparity, n	914 (48.4)	452 (55.6)	511 (61.2)	303 (64.7)	< 0.001
Hypertension, n	10 (0.5)	7 (0.9)	18 (2.2)	8 (1.7)	0.001
Diabetes, n	17 (0.9)	21 (2.6)	29 (3.5)	16 (3.4)	< 0.001
**Adverse pregnancy outcomes**					
Preterm birth	918 (48.6)	302 (37.1)	371 (44.4)	280 (59.8)	< 0.001
Preeclampsia	59 (3.1)	53 (6.5)	93 (11.1)	112 (23.9)	< 0.001
FGR	304 (16.1)	145 (17.8)	176 (21.1)	144 (30.8)	< 0.001
IUFD	57 (3)	12 (1.5)	17 (2)	18 (3.8)	0.026
**Number of adverse pregnancy outcomes**					
0	721 (38.2)	364 (44.8)	322 (38.6)	125 (26.7)	
1	906 (48)	320 (39.4)	332 (39.8)	159 (34)	
2	231 (12.2)	103 (12.7)	136 (16.3)	109 (23.3)	
3	28 (1.5)	22 (2.7)	42 (5)	72 (15.4)	
4	2 (0.1)	4 (0.5)	3 (0.4)	3 (0.6)	

*SCr* serum creatinine, *FGR* fetal growth retardation, *IUFD* intrauterine fetal demise.

### Associational patterns of two grouping methods based on different SCr distributions with adverse pregnancy outcomes

**Figure 3:**
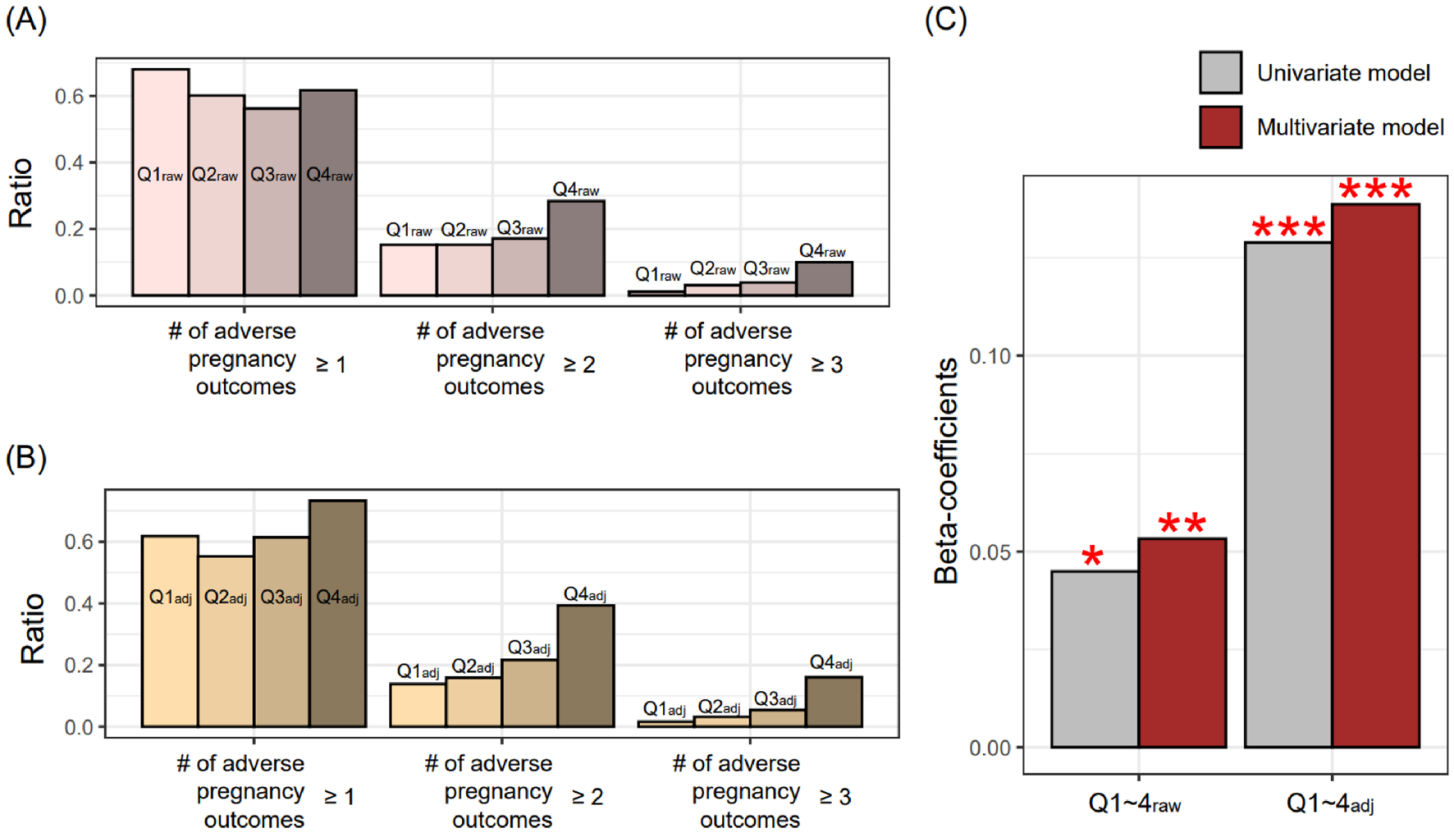
Association between SCr groups and the number of adverse pregnancy outcomes. (**A**,**B**) Adverse pregnancy outcomes include preterm birth, preeclampsia, fetal growth retardation, and intrauterine fetal death; therefore, the maximum value of the number of pregnancy outcomes is four. (**C**) Y-axis indicates beta-coefficients obtained from linear regression with the number of adverse pregnancy outcomes and SCr groups (Q1–4_raw_ or Q1–4_adj_) set as dependent and independent variables, respectively. Age, hypertension, diabetes, and parity types are covariates for multivariate models. *, **, *** indicate *p* value < 0.001, < 0.00001, < 0.0000001, respectively.

The SCr groups stratified based on raw SCr distribution (Q1_raw_–Q4_raw_) could reflect the adverse pregnancy outcomes. In detail, the number of cases with two or more adverse pregnancy outcomes significantly increased from Q1_raw_ to Q4_raw_. However, the ratio of cases with two or more number of adverse pregnancy outcomes increased gradually from Q1_raw_ to Q3_raw_, but drastically from Q3_raw_ to Q4_raw_ (Fig. 3A). The four groups made by the GA-specific SCr distribution showed a more gradual pattern of increase in cases with an adverse pregnancy outcomes (Fig. 3B), compared to the original SCr distribution. For the cases with two or more adverse outcomes, two grouping methods showed a well-characterized stepwise increase pattern (Fig. 3A,B). In uni- or multivariate linear regression (LiR) with SCr group and the number of adverse pregnancy outcomes set as independent and dependent variables, the GA-specific SCr group had high beta-coefficients and low *p*-values, compared to the groups determined by the original SCr distribution (Fig. 3C).

**Figure 4:**
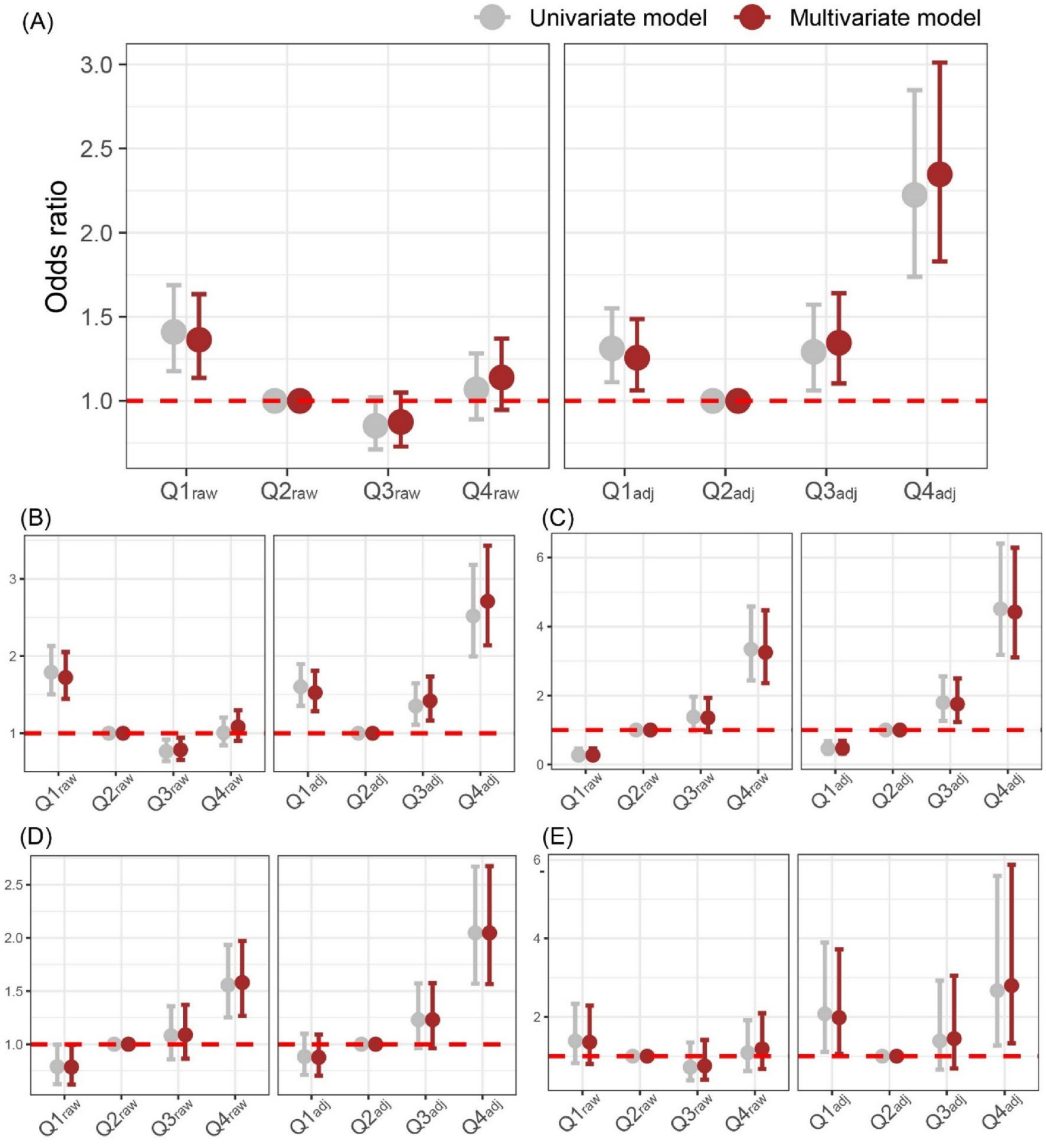
Logistic regression. Q2_raw_ and Q2_adj_ were used as references in all models. Age, hypertension, diabetes, and parity types are covariates for multivariate models. Logistic regression method was used to reveal the relationship between main outcome and four groups based on serum creatinine measurements. Relationship of the four SCr groups with all types of adverse pregnancy outcomes (**A**), preterm birth (**B**), preeclampsia (**C**), fetal growth retardation (**D**), and intrauterine fetal demise (**E**) are illustrated.

We re-grouped all subjects into two groups (with or without an adverse pregnancy outcomes), then conducted uni- or multivariate logistic regression (LR) considering the binary status (adverse pregnancy outcomes vs. control) and quartile group of SCr (Q1–Q4_raw_ or Q1–Q4_adj_) as dependent and independent variables, respectively. Both original and GA-specific SCr distributions had non-linear U shaped associations with adverse pregnancy outcomes, but the results in Q3_raw_ and Q4_raw_ showed insignificant results (Fig. 4A). The GA-specific SCr groups showed a significant U-shaped relationship with the risk for adverse pregnancy outcomes (Fig. 4A). The relational pattern between SCr and PTB was similar with that of all types of adverse pregnancy outcomes (Fig. 4B). In cases of preeclampsia and FGR, the SCr level exhibited a monotonic increasing pattern of relationship (Fig. 4C,D). For intrauterine fetal demise (IUFD), Q1_adj_ and Q4_adj_ had a significantly increased risk (Fig. 4E).

## Discussion

In this retrospective study, we identified that SCr level could predict the risk of adverse pregnancy outcomes as well as the number of co-occurred adverse pregnancy outcomes. Specifically, when using the GA-specific SCr distribution^16^, the predictive power of adverse pregnancy outcomes was more robust based on the beta-coefficients and their *p*-values, compared to the raw SCr distribution (Fig. 3). Moreover, we identified that SCr groups (Q1_adj_–Q4_adj_) determined by the GA-specific SCr distribution exhibited a non-linear U-shaped relationship with the risk of adverse pregnancy outcomes. Park et al.^9^ reported the convergent findings with our results that midterm GFR had a non-linear relationship with the adverse pregnancy outcomes.

Several studies have pinpointed that exclusively using SCr-based equations could result in the misclassification of kidney function during pregnancy^5,16^. To overcome this limitation, Park et al.^9^ only analyzed the midterm GFR values estimated based on SCr levels and their relationship with adverse pregnancy outcomes. Moreover, Harel et al.^16^ collected approximately 362,000 SCr results from about 240,000 women and generated the GA-specific SCr distribution. We could not find the GA-specific GFR distribution and could only obtain the GA-specific SCr distribution^16^, so we designed the linear regression or logistic regression models based on SCr levels, not GFR values. Direct application of the findings obtained from the Canadian population to the Korean pregnant women population may yield inaccurate results due to genetic and ethnic differences. However, we considered this distribution to be generalized, and not biased results because it was obtained from over 360,000 results^16^. In addition, it was obvious to provide an improved prediction of the adverse pregnancy outcomes of Korean women when using the Canadian-based distribution. Future studies to curate Korean-specific SCr distribution based on GA are needed. Moreover, it is crucial to establish the GA-specific GFR distribution.

SCr value is dependent on muscle mass or body composition, such as age, sex, and ethnicity^17^. Due to many factors adversely impacting the accurate estimation of GFR, Kidney Disease Improving Global Outcomes (KDIGO) guidelines recommend confirming CKD status in a specific population initially determined based on GFR estimated by SCr (eGFR_Cr_) using an alternative method, such as GFR estimated by serum cystatin C (eGFR_cys)_^18^. Our study also focused on the inaccurate estimation of kidney function measured by SCr in a specific population (ie, pregnant women), and suggested that the combined information of SCr level and gestational age-specific SCr distribution^16^ could improve the prediction of adverse pregnancy outcomes. In pregnancy, albeit kidney hyperfiltration, the maternal serum level of cystatin C is known to be more stable than SCr before the third trimester^19^. Serum level of cystatin C is known to be elevated in the third trimester of pregnancy; however, its underlying mechanism is not converged^19–21^. In general population, cumulative evidence has supported that eGFR_cys_ is a good alternative method for the GFR estimation, specifically in subgroup exhibiting low muscle amount: elderly population, vegetarians, and those with muscle wasting, chronic disease, or limb amputation^22^. Moreover, most studies commonly suggested that a combined creatinine and cystatin C equation proposed more accurate estimates and greater precision than GFR estimated from creatinine or cystatin C alone^22,23^. Taken together, we suggest that the kidney function of pregnant women is to be measured via multiple serum indices, such as creatinine and cystatin C.

Adverse pregnancy outcomes, such as preeclampsia and PTB, have been considered the leading causes of perinatal morbidity and mortality worldwide^24,25^. Therefore, establishing a prediction model for adverse pregnancy outcomes is crucial to help minimize adverse perinatal outcomes^26,27^. The establishment of the prediction model included two main tasks: one is the feature selection and the other is the optimization of parameters using several machine learning methods^28^. The present study focused on feature selection. Moreover, our results were obtained from previously validated findings^16^, also called prior knowledge, indicating that our study analyzed data based on the Bayesian approach or transfer learning^29^. Our findings indicated a non-linear relationship between SCr levels and adverse pregnancy outcomes; therefore, machine learning methods (e.g., support vector machine, random forest, or deep learning)^30^ that can deal with data exhibiting characteristics of the non-linear or complex relationships among features^30^ are needed to screen patients with high risk adverse pregnancy outcomes.

In the present study, multidisciplinary experts (i.e., laboratorian, database administrator, obstetrician, and computer scientist) performed multiple tasks to construct a database to identify the risk factors for adverse pregnancy outcomes. Initially, obstetricians are responsible for the literature-based review to select candidate features. Later, laboratorian, database administrator, and computer scientist constructed an initial dataset using the automatic platform, including the candidate variables. Then, the obstetrician and laboratorian manually updated the patient’s medical history and pregnancy outcomes.

This study has several limitations. First, because the WSCH is a tertiary hospital, the prevalence of pregnant women with adverse pregnancy outcomes is high compared to general population. To obtain generalized findings, the multi-institutional study or registry is needed, and this study could motivate the multi-center approach. Second, several biomarkers could not be analyzed since the database (e.g., electronic health records and automatic platform) is not designed to compile them. For example, we could not analyze data, such as body mass index (BMI) or waist circumference because they were not recorded at the time of initial pregnancy evaluation. Moreover, due to the retrospective design, we could not control the re-examination of blood tests of pregnant women, resulting that most women examined blood only had a time point of measurement (Fig. 1). Future study that is reinforced with these limitations could identify the valuable pathophysiological signatures related to decreased renal function and blunted hyperfiltration, and adverse pregnancy outcomes.

We implemented prior knowledge^6^ obtained from the enormously large scale of data and observed that elevated SCr levels were significantly related with the risk of adverse pregnancy outcomes. This study is a basic task of developing an algorithm to predict the possibility of adverse pregnancy outcomes based on the pregnant women's SCr levels adjusted by gestational weeks.

## Methods

### Data collection

We performed a retrospective study on pregnant women giving birth between January 2010 and December 2020 at the WSCH. Eligible subjects were women who had a singleton birth at more than 20 weeks gestation and were aged 16–50 years at the time of delivery. We constructed the initial dataset with the above criterion (i.e., 20 weeks and age) by database administrator as our previous work^29^. Afterward, we selected pregnancy cases based on patients’ case notes that had been manually recorded by the department of Obstetrics and Gynecology. We excluded pregnant women that did not have data for SCr levels and SCr measurement times.

The present study was approved by the Institutional Review Boards (IRB) of WSCH (CR321084). This study was conducted in accordance with the principles of the Declaration of Helsinki. The study was an observational study without medical intervention, so the need to obtain informed consent from patients was waived. The waiver of informed consent was also confirmed by the above IRB of WSCH (CR321084).

### Adverse pregnancy outcomes

Several studies have analyzed the association between kidney function and adverse pregnancy outcomes^8,16^. Park et al.^7^ defined pregnancy outcomes as being adverse when a pregnant woman has one or more of the following: PTB, low birth weight, or preeclampsia. Harel et al.^14^ selected extreme PTB, preeclampsia, perinatal mortality, and severe FGR as the main adverse pregnancy outcomes. Motivated by these studies, we determined PTB (delivery before 37 weeks gestation), preeclampsia (blood pressure ≥ 140/90 with significant proteinuria and/or end organ damage), FGR (birthweight below 10th percentile adjusted for gestational age), and IUFD as adverse pregnancy outcomes.

### Covariates

Harel et al.^8^ determined maternal age, rural residence, region of origin, gestational week, preconception SCr, chronic diseases (diabetes and hypertension), and tobacco/illicit drug (each ≤ years before conception) as covariates. Park et al.^7^ selected age, BMI, weight gain until delivery, hypertension, diabetes, hypertensive disorder during pregnancy, previous history of stillbirth/miscarriage, multiparity, and hospitalization status. Motivated by these studies, we categorized the parity type into two groups: nulliparity and multiparity. For underlying maternal diseases, hypertension and diabetes were included in this study, which were determined only by diagnosis code (i.e., ICD10) due to lack of data pertaining to blood pressure or serum fasting glucose at the time of SCr measurement. SCr measurement was performed using the Cobas® 8000 system (Cobas® c 702 and e 601 module; Roche Diagnostics, Switzerland).

### Statistics

Continuous and categorical variables were analyzed according to the four groups categorized by the GA-specific SCr distribution^16^ using one-way analysis of variance (ANOVA) and Chi-square test, respectively. To test for linear trends of categorical or continuous variables, we determined the median SCr levels of each group as continuous variables in the Chi-square test and one-way ANOVA. All statistical analyses and graphic works were performed using R software (version 4.0.1, the R Foundation). A two-sided *p* value less than 0.05 was considered to indicate statistical significance for all analyses.

## Figures and Tables

**Figure 5:**
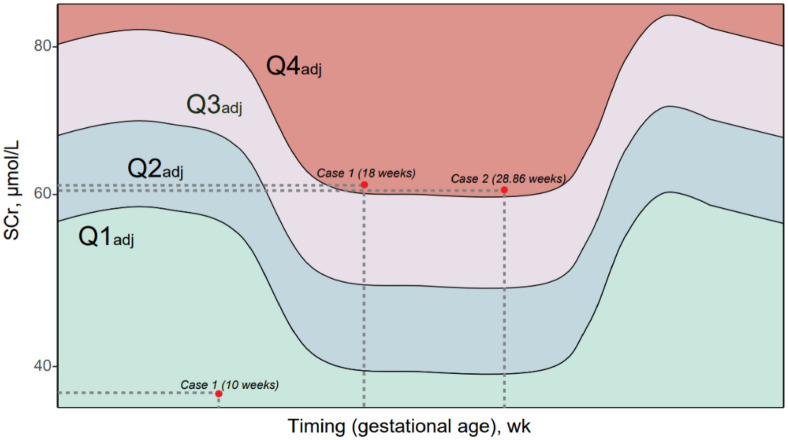
Application of the gestational age-specific serum creatinine distribution^16^ in clinical setting.

## Data Availability

The data that support the findings of this study are available from the corresponding author upon reasonable request.

## Appendix

### Case 1

A 35 years woman visited in the department of Obstetrics for the laboratory tests at initial pregnancy evaluation. She had no medical history such as hypertension, diabetes, and CKD. Her GA was 10 weeks and the SCr level was 26.5 μmol/L (0.3 mg/dL) which is within normal range. She measured SCr level at 18 week of GA, showing normal value of 61.9 μmol/L (0.7 mg/dL). We mapped her SCr levels onto GA-specific SCr distribution made by Harel et al.^16^, and found that the SCr levels were located in Q1 and Q4 (Fig. 5) indicating a high risk of decreased renal function or blunted hyperfiltration in 18 weeks. She finally exhibited two cases of adverse pregnancy outcomes, including PTB and FGR.

### Case 2

A 28 years women visited WSCH for the laboratory tests at the first prenatal encounter. She had no medical history. Her GA was 28.86 weeks and the SCr was measured once and its level was 60.996 μmol/L (0.69 mg/dL) that is normal range. After mapping it onto the GA-specific SCr distribution^16^, it was located in Q4 (Fig. 5). She finally resulted three cases of adverse pregnancy outcomes, including PTB, preeclampsia, and FGR.
